# Regulation of neutrophil migration in acute pulmonary inflammation by extraneuronal α1 gamma-aminobutyric acid_A_ receptors

**DOI:** 10.1038/s41419-025-07488-1

**Published:** 2025-04-18

**Authors:** Anika Fuhr, Robin Roediger, Mariana Simelitidis, Jutta Gamper-Tsigaras, Markus Templin, Michael Kormann, Bernd Antkowiak, Uwe Rudolph, David Köhler, Peter Rosenberger, Kristian-Christos Ngamsri, Franziska M. Konrad

**Affiliations:** 1 Department of Anesthesiology and Intensive Care Medicine, University Hospital of Tuebingen, Tübingen, Germany; 2 NMI Natural and Medical Sciences Institute, University Tuebingen, Tübingen, Germany; 3 Stem Cell Network Tuebingen, University Tuebingen, Tübingen, Germany; 4 Department of Comparative Biosicences, College of Veterinary Medicine, and Carl R. Woese Institute for Genomic Biology, University of Illinois Urbana-Champaign, Champaign, IL, USA

**Keywords:** Acute inflammation, Innate immunity

## Abstract

Acute pulmonary inflammation is a common disease on intensive care. Due to the lack of specific treatments, lethality is still very high. The ionotropic GABA_A-_receptors are known from the central nervous system (CNS) and have recently been detected in the lung. These receptors have been shown to influence inflammatory processes. Opposing data has been reported, where both, GABA site agonists and antagonists achieved anti-inflammatory effects. The distribution of the 19 known GABA_A-_receptor subunits (α1-6, β1-3, γ1-3, δ, ε, π, θ and ρ1-3) and their role in inflammation remain unclear. In murine models of LPS- and bacteria-induced inflammation, Muscimol (GABA_A_-receptor agonist) and Bicuculline (antagonist) were administered before the onset of inflammation. Transcription of GABA_A_-receptor subunits was evaluated by real-time polymerase chain reaction. Neutrophil counts and adhesion molecule expression in wild type and GABAα1 knock-in mice were determined by flow-cytometry. Myeloperoxidase, neutrophil extracellular traps and cytokines were determined. In a model of ventilator-induced lung injury, blood gas analysis was performed using arterial blood. A multiplex western blot was done to assess signaling proteins. Muscimol and Bicuculline inhibited neutrophil influx in the bronchoalveolar lavage but did not change neutrophil activation. Both altered surface expression of adhesion molecules on neutrophils and reduced release of interleukin-6 (IL-6). The increased α1 subunit expression on lung epithelium and endothelium after inflammation was abolished by Muscimol and Bicuculline. In GABAα1-knock-in mice the protective effects of both agents were no longer observed. Only Muscimol lowered protein extravasation, improved blood gas analysis and lung function. A multiplex assay ascribed these anti-inflammatory effects to the influence of the IL-6 and phosphoinositide 3-kinase signaling pathways. In conclusion, Muscimol and Bicuculline exert various protective effects in two murine models of acute pulmonary inflammation. The multiple effects of Muscimol were linked to the inhibition of the proinflammatory signaling pathways IL-6 and PI3K.

## Introduction

The Acute Respiratory Distress Syndrome (ARDS) is a common disease on intensive care units with an incidence of over 10% of all intensive care patients [1]. Because of its high lethality, it is a very dreaded disease and there are no specific therapies available yet [1].

ARDS has various causes and yet a unified cascade of pathophysiologic mechanisms is activated consistently. This includes the damage of the alveolar-capillary barrier and the migration of neutrophils into the alveolar space [2].

Studies have shown that lung microvascular endothelial cells, alveolar epithelial cells type 2 and neutrophils express gamma-aminobutyric acid (GABA)_A_-receptors, which are widely distributed in the central nervous system (CNS) [3–6]. The GABA_A_-receptors are assembled from a repertoire of 19 subunit isoforms (α1-6, β1-3, γ1-3, δ, ε, π, θ and ρ1-3) and the most common receptors contain two α-subunits, two β-subunits and a γ-subunit [7, 8].

The GABAergic system regulates fluid homeostasis and innate immune responses. Migration of immune cells can be reduced by the inhibited release of interleukin-6 (IL-6) triggered by the activation of GABA-receptors [9]. Activated GABA_A_ receptors are responsible for a Cl^-^ efflux and membrane depolarization in endothelial and epithelial cells, leading to intercellular gaps and thereby edema in inflammation [10, 11]. Functions of GABA_A_-receptors vary with the composition of different subunits. A proinflammatory role of the α3-subunit was described, while activation of α1-, α4- and α5-subunits decreased inflammation [12]. Several studies evaluated the expression of GABA_A_ receptor subunits in mouse and rat lungs and depicted a high expression of the α1 subunit in adult animals [13, 14]. This suggests that the GABAα1 subunit plays a significant role in acute pulmonary inflammation and ARDS. Muscimol, a GABA_A_-receptor agonist, binds to GABA_A_ receptors at the GABA binding site, located between the α and β subunit. Muscimol was shown to improve survival and inhibited inflammation in LPS-treated mice [15]. But likewise, there are studies proving that inhibition of the GABA_A_-receptor has an anti-inflammatory effect [16–18]. Bicuculline is a GABA_A_-receptor antagonist whose binding site is also located between the α and β subunit, which has also been shown to have anti-inflammatory effects [18, 19].

In the present study, we investigated the role of pulmonary GABA_A_-receptors, specifically of the α1 GABA_A_-receptor and its influence on neutrophil migration. We investigated the effects of Muscimol and Bicuculline on neutrophil influx, chemokine release, blood gas analysis and microvascular permeability in acute pulmonary inflammation.

## Results

### Gene and protein expression of GABA_A_-receptor subunits in pulmonary inflammation

**Fig. 1:**
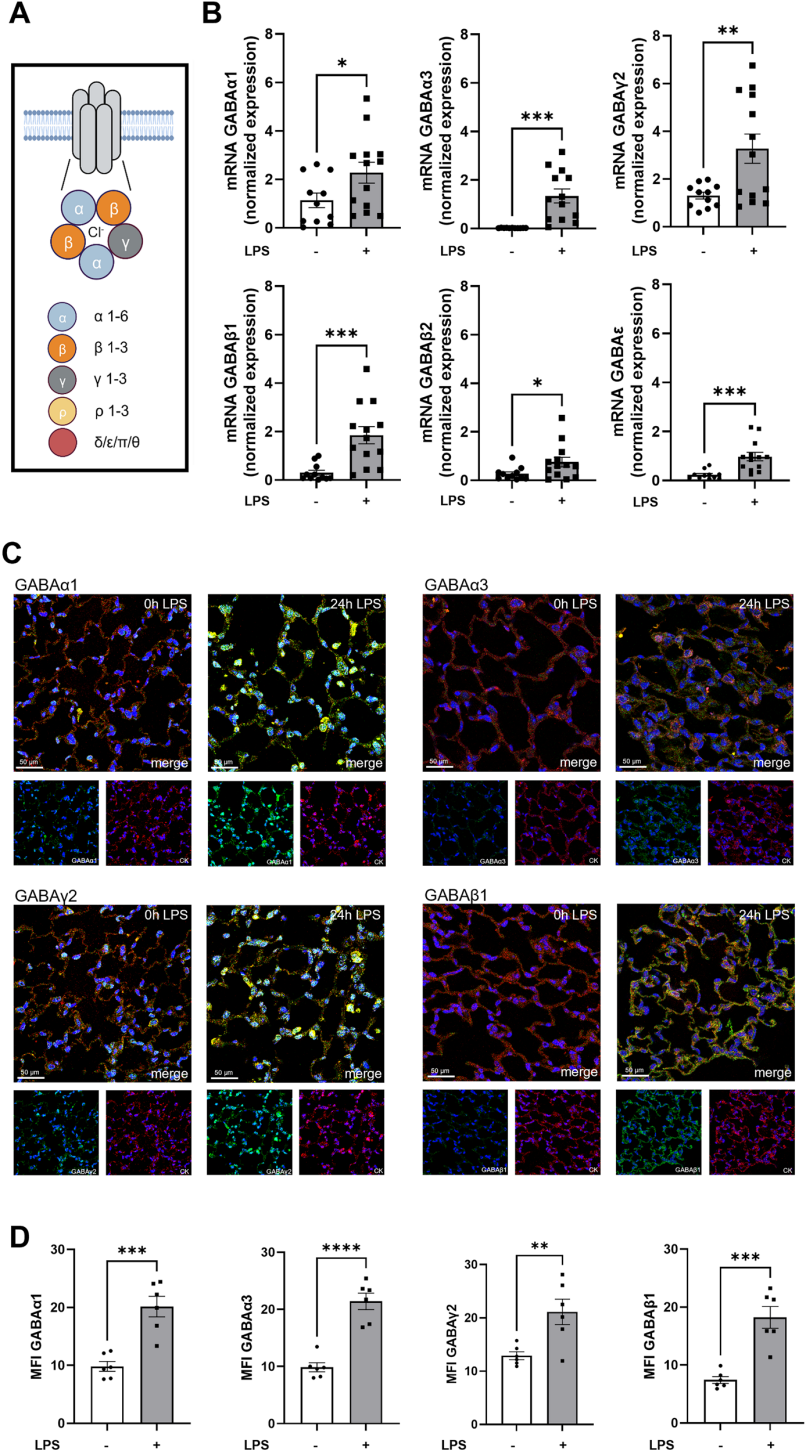
Expression of GABA_A_-receptor subunits during acute pulmonary inflammation. LPS-induced pulmonary inflammation increased expression of different GABA_A_-receptor subunits on gene and protein level. **A** Schematic representation of the heteropentameric form of the transmembrane GABA_A_-receptor in its most abundant form with two α, two β and one γ subunit. Created with biorender.com. **B** Four hours after LPS inhalation, mRNA expression of GABA_A_-receptor subunits α1, α3, γ2, β1, β2 and ε was determined by quantitative real-time polymerase chain reaction. **C** Identification of GABA_A_-receptor subunit expression of the subunits α1, α3, γ2 and β1 (all appear in green) in lung tissue samples (Images are representative slides of *n* = 3 independent experiments; 20x magnification). Lung architecture was visualized by staining the cytoskeleton of epithelial cells (appears red) and counterstaining of the nuclei was performed using DAPI (appears blue). Lung tissue samples without and 24 hours after LPS inhalation were compared. **D** Mean fluorescence intensity (MFI) of the GABA_A_-receptor subunits in lung tissue was quantified using LAS X (*n* = 6). The data are presented as mean ± standard error of mean (SEM). *P < 0.05, **P < 0.01, ***P < 0.001, ****P < 0.0001. Statistical analyses were performed by unpaired *t*-tests.

GABA_A_-receptors are built from a pool of 19 subunits with the most common form consisting of two α, two β and one γ subunit (Fig. 1A). The composition of the GABA_A_-receptors in the lung and their role in inflammation is still unclear.

Based on the current study situation, we investigated the GABA_A_-receptor subunits α1, α3-5, β1-3, γ2, γ3 and ε (data for α4, α5, β3 and γ3 due to non-existent alterations by LPS, Muscimol and bicuculline is not shown) [20, 21]. In wild type mice, the α1- and γ2-subunit showed the highest gene expression levels under physiological conditions in lung tissue. LPS caused a significant increase of α1, α3, β1, β2, γ2 and ε (Fig. 1B, statistical evaluation see Supplementary Information (SI) 1.

Immunofluorescence staining of the GABA_A_ subunits α1, α3, β1 and γ2 in lung slices of wild type mice was performed (Fig. 1C) and the mean fluorescence intensity (MFI) quantified (Fig. 1D). MFI of all four subunits was significantly increased after LPS inhalation.

### Inflammation changes the composition of GABA_A_-receptors in the lung

**Fig. 2:**
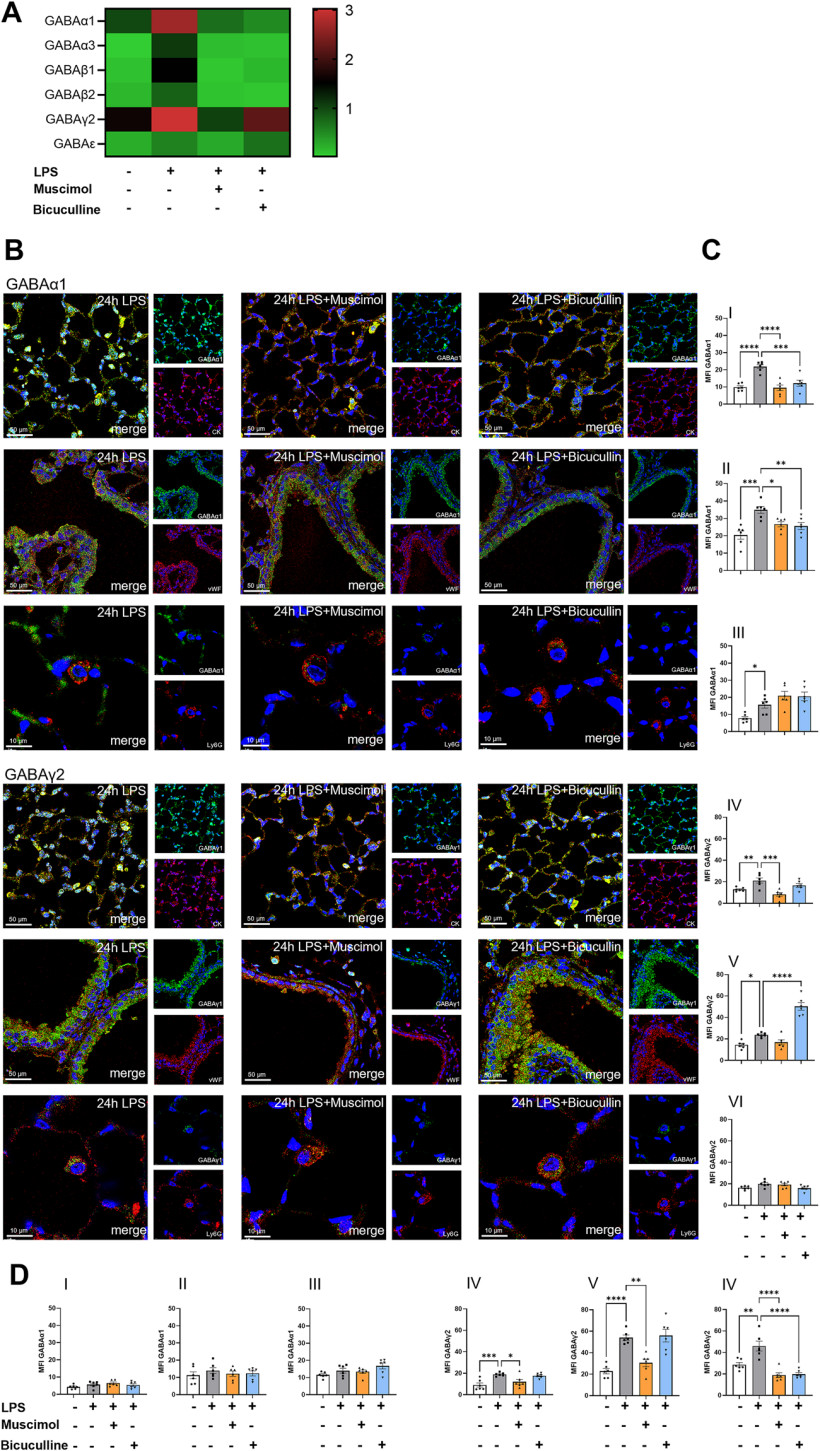
Effect of Muscimol and Bicuculline on gene and protein expression levels of GABA_A_-receptor subunits. Muscimol treatment decreased the expression of all GABA_A_-receptor subunits studied, whereas Bicuculline showed no effect for γ2 and ε. **A** The transcriptional data for GABA_A_-receptor subunit expression in lung tissue four hours after LPS-derived inflammation is shown. Each row displays the normalized gene expression of one subunit tested and each column represents a treatment group. The expression level is color-coded from red (upregulated) to black to green (downregulated) (*n* ≥ 7). **B** At the protein level, immunofluorescence was used to investigate in detail on which cell type Muscimol and Bicuculline exert their effect on the expression of GABAα1 and GABAγ2 24 hours after LPS-inhalation. The subunits are each shown in green. Specific staining of epithelial cells (CK), endothelial cells (vWF), and neutrophils (Ly6G) is shown in red. Counterstaining of the nuclei was performed using DAPI and is shown in blue. Images are representative slides of *n* = 3 independent experiments; 63x magnification. **C** MFI of GABAα1 (I-III) and GABAγ2 (IV-VI) in wild type mice was quantified using LAS X (*n* = 6). The expression of the subunits was specifically investigated on epithelial cells (I, IV), endothelial cells (II, V) and neutrophils (III, VI). **D** Quantified MFI of GABAα1 (I-III) and GABAγ2 (IV-VI) in GABAα1-Knock-in mice from immunofluorescence staining on epithelial cells (I, IV), endothelial cells (II, V) and neutrophils (III, VI) (Staining shown in SDC 3). Data are presented as mean ± SEM. *P < 0.05, **P < 0.01, ***P < 0.001, ****P < 0.0001. One-way ANOVA and Bonferroni correction was used for multiple group comparison.

We used Muscimol and Bicuculline to determine the impact of GABA_A_-receptors. On the transcriptional level, both suppressed the subunits α1, α3, β1 and β2, but for γ2 and ε, only Muscimol decreased gene levels significantly (Fig. 2A). GABAα1 and GABAγ2 were further evaluated on lung epithelium, endothelium and neutrophils (Fig. 2B). Statistical evaluation is shown in Fig. 2C. Muscimol and Bicuculline significantly decreased the protein expression of GABAα1 on the lung epithelium and endothelium of wild type animals, but not on neutrophils. For GABAγ2, Muscimol significantly lowered protein expression only on epithelial tissue. However, Bicuculline increased GABAγ2 protein expression, but only on the endothelium.

The α1 and γ2 subunits were expressed highly in the lung and significantly increased during inflammation. Since α1 is part of the the binding site for Muscimol and Bicuculline, we hypothesized that this subunit is important for their anti-inflammatory effects. Immunofluorescence staining of α1 and γ2 subunits on the epithelium, endothelium and neutrophils was performed in GABAα1-Knock-in mice and statistical evaluation is shown in Fig. 2D (representative images are shown in SI 2). The Knock-in of GABAα1 lowered α1 subunit expression on all cell types and was not affected by LPS, nor by Muscimol and Bicuculline. LPS triggered a significantly increased protein expression of γ2 subunit in GABAα1-Knock-in mice, whereas Muscimol significantly inhibited the GABAγ2 level. Bicuculline had a selective suppressive effect on γ2 expression on neutrophils.

### Muscimol and Bicuculline have α1 subunit-dependent anti-inflammatory effects on neutrophil migration in vivo and in vitro

**Fig. 3:**
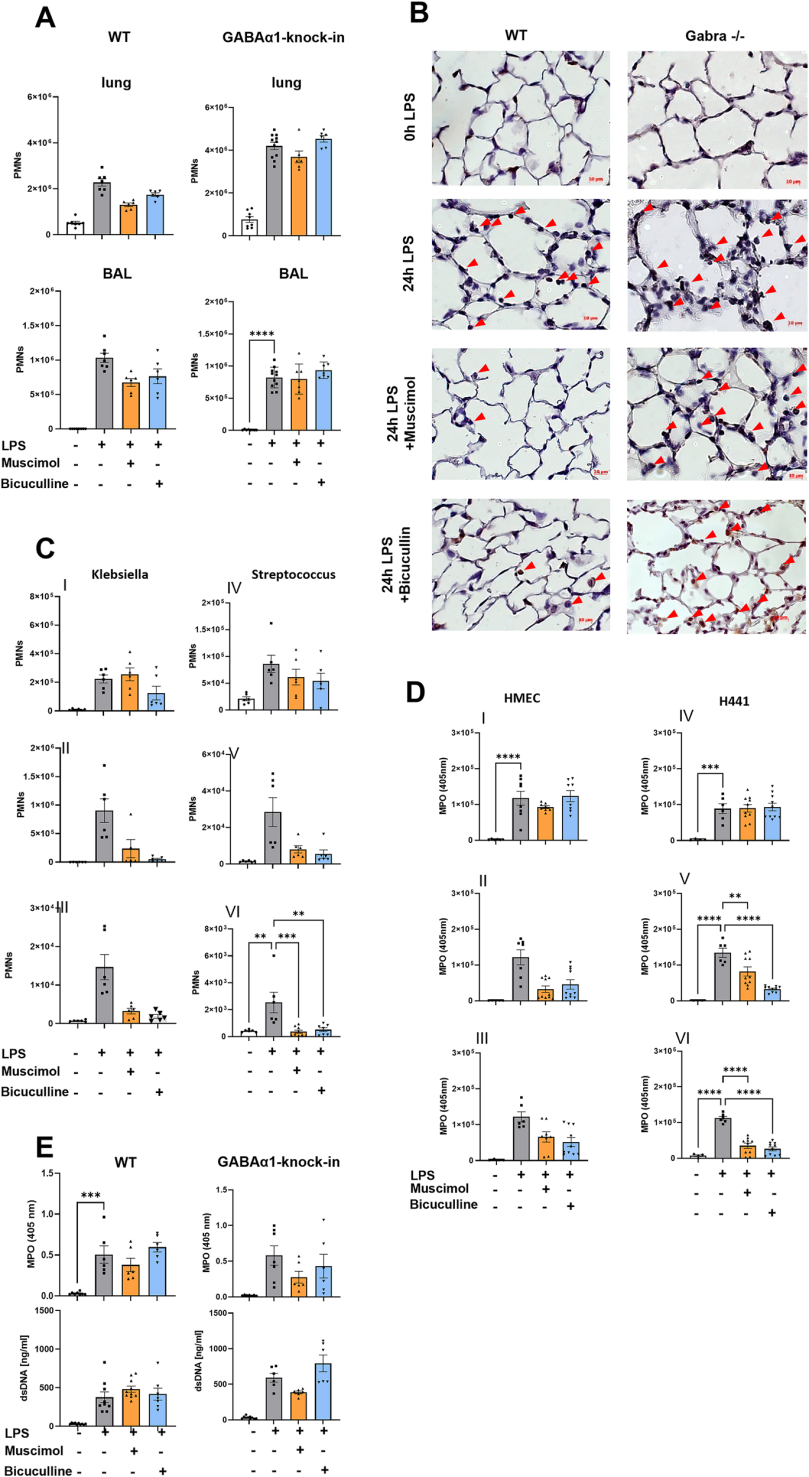
Muscimol and bicuculline affect migration but not activation of neutrophils in an α1 receptor subunit-dependent manner. **A** neutrophil accumulation into the lung and bronchoalveolar lavage (BAL) was assessed by flow cytometry in wild type and GABAα1-Knock-in mice without and with LPS induced inflammation, also with treatment by Muscimol and Bicuculline (*n* ≥ 6). **B** Immunohistochemical staining was performed with lung sections (*n* = 4). Neutrophils were marked specifically by an antibody and appear brown, hematoxylin was used for counterstaining. **C** The migration of neutrophils was further studied in more detail in a model with inflammation induced by Klebsiella pneumoniae (I-III) or Streptococcus pneumoniae (IV-VI) in wild type mice (*n* ≥ 6). Here, a distinction was made between neutrophils adherent to the endothelium (I, IV), located in the interstitium (II, V), and migrated into the alveolar space (III, VI). **D** Migration of neutrophils was mimicked in an in vitro system in which freshly isolated human neutrophils migrate along a chemokine gradient through a monolayer of endothelial cells (HMEC; I-III) or epithelial cells (H441; IV-VI) (*n* ≥ 7 in three independent experiments). Three different settings of the experiment were conducted. Firstly, only the monolayer was treated with Muscimol or Bicuculline (I, IV), secondly, only the neutrophils were treated (II, V) and in the third part, both cell types in the system were treated (III, VI). **E** The activity of neutrophils was measured using a myeloperoxidase (MPO) assay and quantification of double-stranded DNA (dsDNA) from the supernatant of BAL 24 hours after LPS inhalation in wild type and GABAα1-Knock-in mice (*n* ≥ 6). MPO is an enzyme secreted by neutrophils after their activation and dsDNA indicates the NETosis activity of neutrophils. Data are presented as mean ± SEM. *P < 0.05, **P < 0.01, ***P < 0.001, ****P < 0.0001. One-way ANOVA and Bonferroni correction was used for multiple group comparison.

To identify the impact of Muscimol and Bicuculline on neutrophil migration, neutrophils were evaluated by flow-cytometry (Fig. 3A). In wild type mice, LPS increased neutrophil counts in the lung and BAL significantly. Muscimol and Bicuculline significantly decreased neutrophil-influx into both compartments. In GABAα1-Knock-in mice LPS also increased neutrophil migration into the lung and BAL, but the anti-inflammatory effects of Muscimol and Bicuculline were abolished.

To visualize the flow-cytometry based data, immunohistochemical staining was performed with mice lung sections (Fig. 3B). In wild type mice, LPS led to a neutrophil influx, Muscimol and Bicuculline reduced neutrophil migration. In GABAα1-Knock-in mice, LPS also raised neutrophil counts but showed no changes after Muscimol and Bicuculline.

To verify whether the LPS-related results apply to other clinically relevant bacterial pathogens, wild type animals were exposed to gram-negative respectively gram-positive bacteria (Fig. 3C). In addition, antibody staining was extended to observe which migratory step is affected by the modulation of the GABA_A_-receptor. Neutrophil counts increased significantly in all three lung compartments. In the interstitium and BAL, a significant decrease of neutrophil counts after both treatments was detected. These results show that transendothelial and transepithelial migration were addressed.

We verified our murine in vivo migration results by in vitro transmigration assays. Human neutrophils were stimulated with fMLP to migrate through a monolayer of endothelial (Fig. 3D I-III) respectively epithelial cells (Fig. 3D IV-VI). Stimulation caused significantly more migrated neutrophils. GABA_A_-receptor modulation on endothelial and epithelial (I and IV) cells had no effect on the transmigration, but treatment of neutrophils (II and V) led to a significantly decreased migration. Treatment of both (III and VI), monolayer and neutrophils showed an additive effect.

To evaluate the impact of GABA_A_-receptor modulation on neutrophil activation, we measured the concentration of MPO in murine BAL (Fig. 3E). Both, wild type, and GABAα1-Knock-in mice showed an increase of MPO level after LPS, but no significant decrease after Muscimol or Bicuculline. Further, we determined the concentration of dsDNA as a marker for NETosis (Fig. 3E). The determination of dsDNA confirmed the MPO data.

### Surface expression of adhesion molecules on neutrophils changes after Muscimol and Bicuculline

**Fig. 4:**
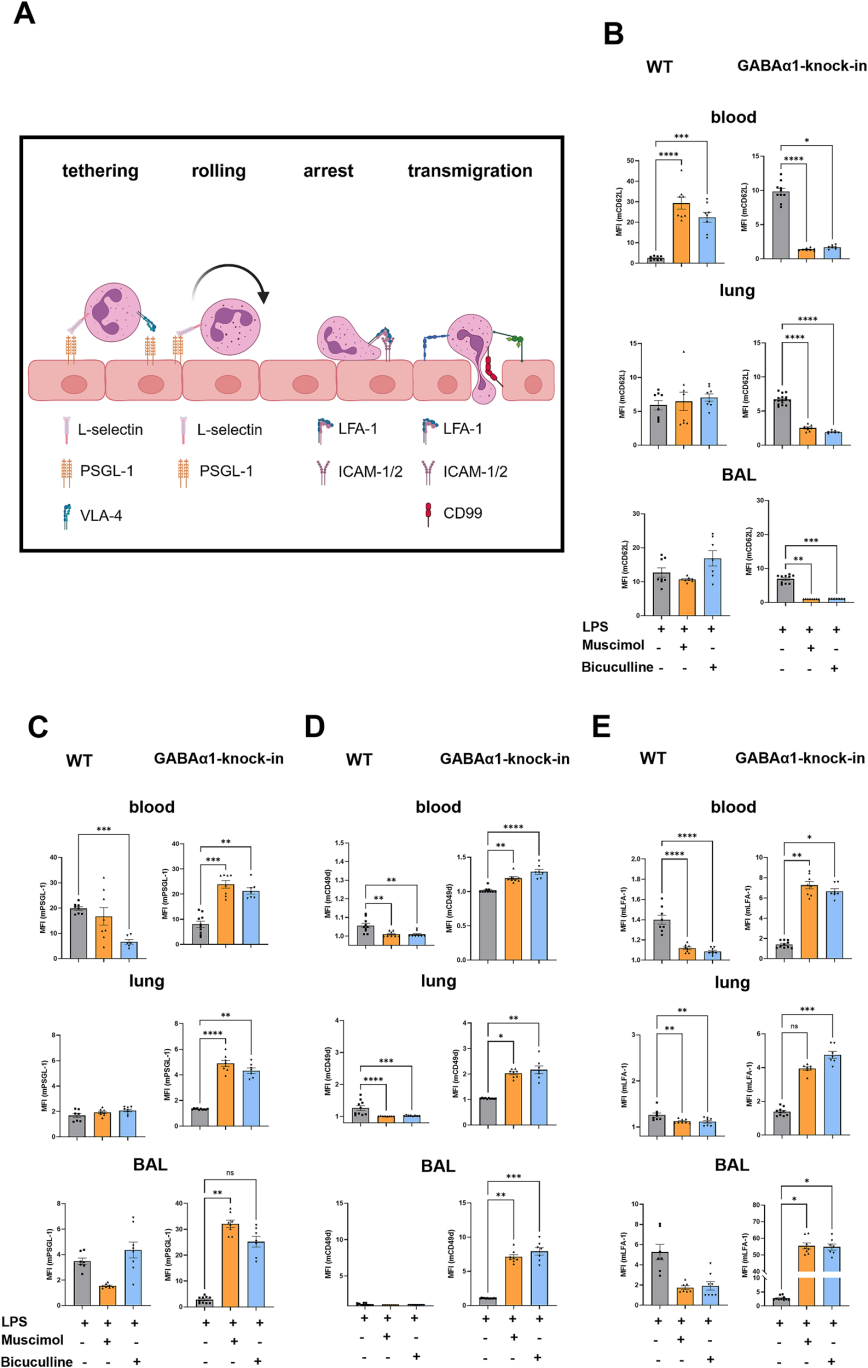
Agonistic and antagonistic regulation of the receptor differentially alters the expression of adhesion molecules in wild type and GABAα1-knock-in animals. **A** Schematic representation of the individual steps of neutrophil migration with the associated adhesion molecules L-selectin, P-selectin glycoprotein ligand-1 (PSGL-1), very late antigen-4 (VLA-4), lymphocyte function-associated antigen-1 (LFA-1), intercellular adhesion molecule-1/2 (ICAM-1/2) and cluster of differentiation 99 (CD99). Created with biorender.com. **B**–**E** The mean fluorescence intensity of the measured adhesion molecules was quantified by flow cytometry. Blood, lung, and BAL from wild type and GABAα1-Knock-in animals were analyzed 24 hours after inducing inflammation by LPS. The following were examined: **B** CD62L, **C** PSGL-1, **D** CD49d, and **E** LFA-1. Data are presented as mean ± SEM. *P < 0.05, **P < 0.01, ***P < 0.001, ****P < 0.0001. One-way ANOVA and Bonferroni correction was used for multiple group comparison.

Transendothelial migration consists of several substeps: first tethering, followed by rolling, arrest and transmigration (Fig. 4A).

We investigated the surface expression of adhesion molecules on neutrophils relevant for migration [22]. In the blood of wild type mice, L-Selectin was significantly increased after GABA_A_-receptor modulation, whereas treatment in GABAα1-Knock-in mice significantly downregulated L-Selectin on neutrophil surface (Fig. 4B). Significantly less PSGL-1 was found on the neutrophils in the blood of wild type animals with Bicuculline treatment. In the GABAα1-Knock-in animals, PSGL-1 was upregulated by Muscimol and Bicuculline in all three compartments (Fig. 4C).

CD49d (Fig. 4D), the alpha chain of the integrin Very Late Antigen-4 (VLA-4) and Lymphocyte Function Associated Antigen-1 (LFA-1) (Fig. 4E) were significantly less expressed in the blood and lungs due to GABA_A_-receptor modulation. In the GABAα1-Knock-in mice, both integrins were significantly increased on the surface due to the receptor modulation.

### Influence of GABA_A_-receptor modulation on the release of chemokines

**Fig. 5:**
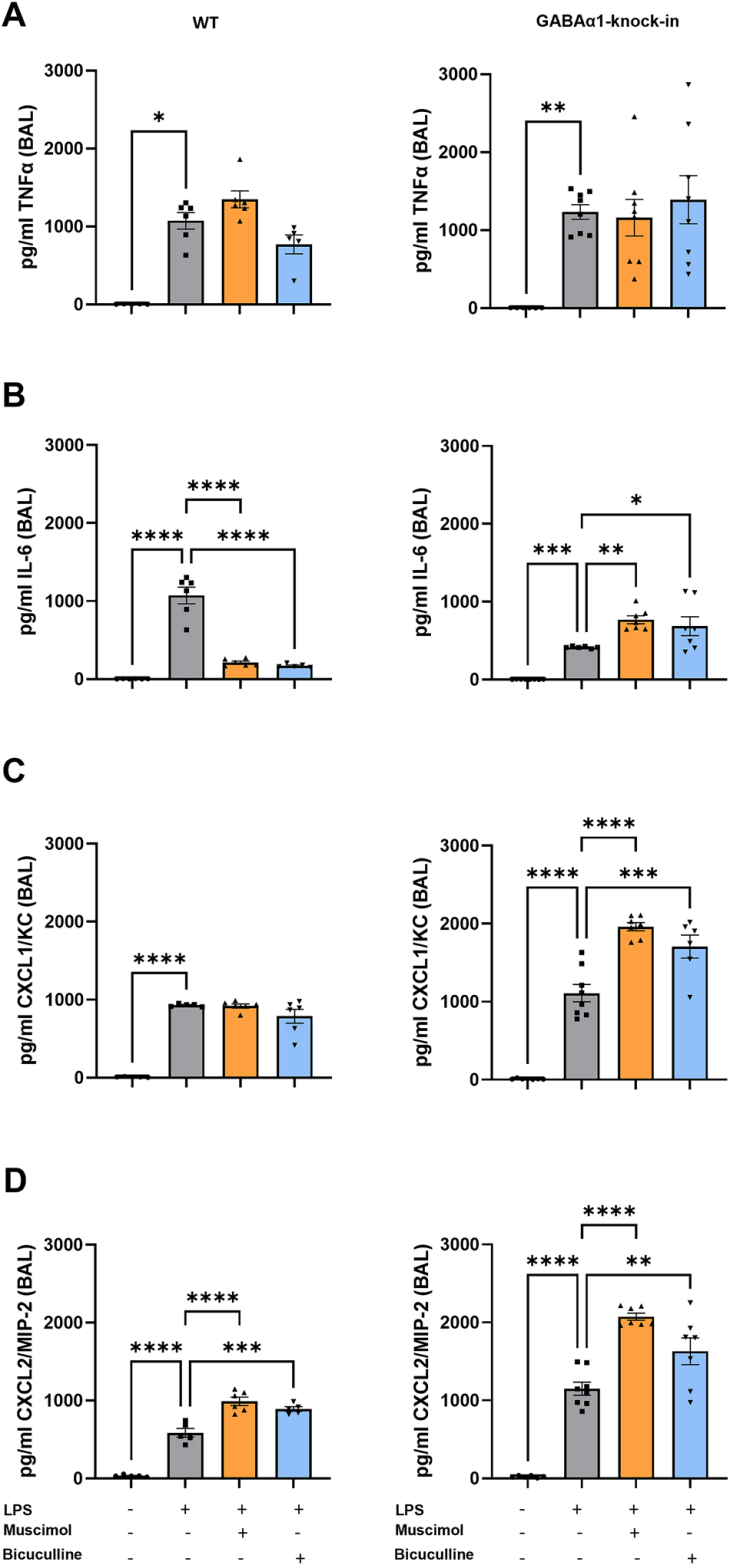
Release of inflammatory mediators is changed by modulation of the GABA_A_-receptor. The release of cytokines was quantified by enzyme-linked immunosorbent assay (ELISA). The samples used were the supernatants of BAL from wild type and GABAα1-Knock-in animals, 3 hours after LPS inhalation (*n* ≥ 6). The investigations focused on the two proinflammatory cytokines (**A**) tumor necrosis factor α (TNFα) and (**B**) interleukin-6 (IL-6), as well as the chemokines (**C**) keratinocyte-derived chemokine (CXCL1/KC) and (**D**) CXCL2/3/MIP-2 macrophage inflammatory protein-2 (CXCL2/3/MIP-2). Data are presented as mean ± SEM. *P < 0.05, **P < 0.01, ***P < 0.001, ****P < 0.0001. One-way ANOVA and Bonferroni correction was used for multiple group comparison.

In wild type and GABAα1-Knock-in mice, LPS increased TNFα (Fig. 5A) and IL-6 (Fig. 5B) concentration in the BAL. Muscimol and Bicuculline did not affect the release of TNFα. In contrast, both agents led to a significantly lower IL-6 release, and a significantly increased IL-6 concentration in GABAα1-Knock-in mice. For CXCL1/KC (Fig. 5C) and CXCL2/3/MIP-2 (Fig. 5D), LPS increased levels in wild type and GABAα1-Knock-in mice but were not affected by both agents.

### Muscimol but not Bicuculline ameliorates microvascular leakage

**Fig. 6:**
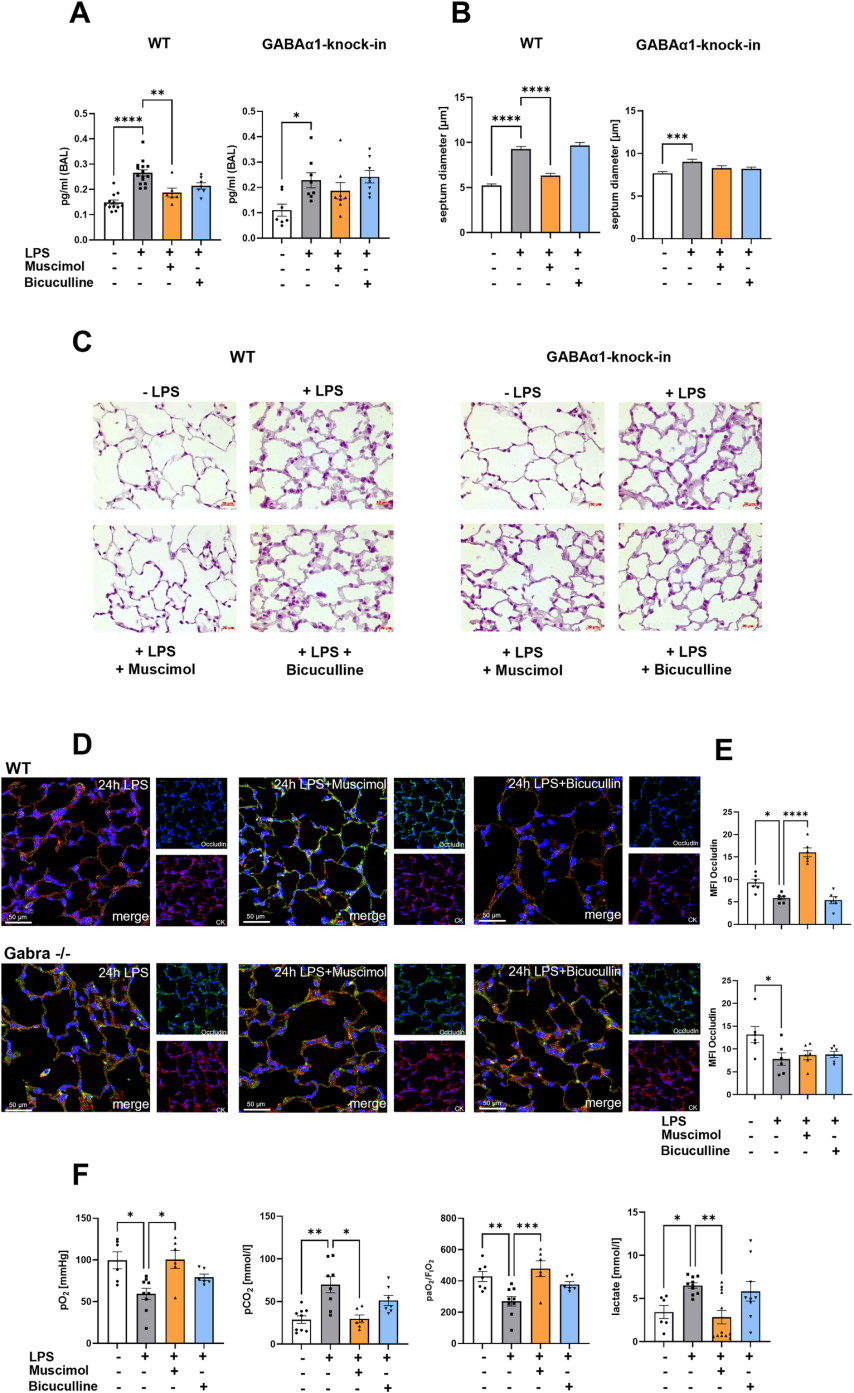
Muscimol plays a significant α1 subunit-dependent role on microvascular permeability and gas exchange, whereas Bicuculline has no effect on it. **A** To investigate microvascular permeability, total protein levels proportional to permeability damage were quantified in the BAL using a bicichinoninic acid (BCA) assay (*n* ≥ 6). The measurement was performed in the supernatants of BAL 3 hours after the onset of inflammation by LPS inhalation in wild type and GABAα1-Knock-in animals. **B** Alveolar septum thickness was measured in (**C**) histological images with mayer’s hematoxylin of lung tissue from wild type and GABAα1-Knock-in animals 24 hours after inflammation onset. The measurement was performed using ImageJ and 5-7 septa were measured per section in each of 5 representative image sections (*n* ≥ 25). **D** Immunofluorescence staining was also performed with the lung sections to show how the cell-cell junctions change. The adhesion molecule occludin appears green. In addition, the cytoskeleton of the cells was stained (red) and DAPI was used as counterstain of the nuclei (blue). Images are representative slides of *n* = 3 independent experiments; 20× magnification. **E** LAS X was used to quantify the mean fluorescence intensity of occludin in lung tissue (*n* = 6). **F** Arterial blood was obtained by puncture of the left ventricle of wild type animals for blood gas analysis. Oxygen partial pressure, carbon dioxide partial pressure, Horovitz quotient and lactate were measured four hours after LPS inhalation. Data are presented as mean ± SEM. *P < 0.05, **P < 0.01, ***P < 0.001, ****P < 0.0001. One-way ANOVA and Bonferroni correction was used for multiple group comparison.

Permeability was assessed by measuring the total protein concentration in the BAL (Fig. 6A). Inflammation increased the protein concentration significantly. This was abolished by Muscimol in wild type mice. In GABAα1-Knock-in mice, neither Muscimol, nor Bicuculline had significant effects. To confirm the data, changes in alveolar septum diameter were determined (Fig. 6B) in mice lung sections (Fig. 6C). LPS thickened alveolar walls, but Muscimol significantly restored the damage in wild type animals. Bicuculline, again, had no effect. In GABAα1-Knock-in mice, both GABA_A_-receptor treatments had no impact on lung tissue damage. To further determine the effects of Muscimol and Bicuculline on pulmonary barrier function, we evaluated their influences on the expression of the tight junction protein occludin (Fig. 6D). In wild type mice, occludin was significantly reduced after LPS (Fig. 6E). Only Muscimol abolished this effect. In GABAα1-Knock-in animals, LPS also reduced occludin, and Muscimol and Bicuculline did not affect it.

In blood gas analysis (Fig. 6F) The arterial oxygen partial pressure (paO_2_) decreased with inflammation, but significantly elevated after Muscimol. The arterial carbon dioxide partial pressure (paCO_2_) was increased after LPS-inhalation and significantly lowered after Muscimol. The Horovitz index, as a parameter for lung function, decreased after LPS and improved significantly after Muscimol.

Lactate level is an indicator of the oxygen supply to the organs. Our results showed a significant increase of lactate after LPS and a significant drop with Muscimol.

### Muscimol alters signaling pathways in acute pulmonary inflammation

Muscimol showed anti-inflammatory effects on neutrophil migration, chemokine release and permeability, whereas Bicuculline had no protective effect on permeability. Since Muscimol has shown more potential as a treatment option due to its multi-faceted effects, we focused on Muscimol in the following.

**Fig. 7:**
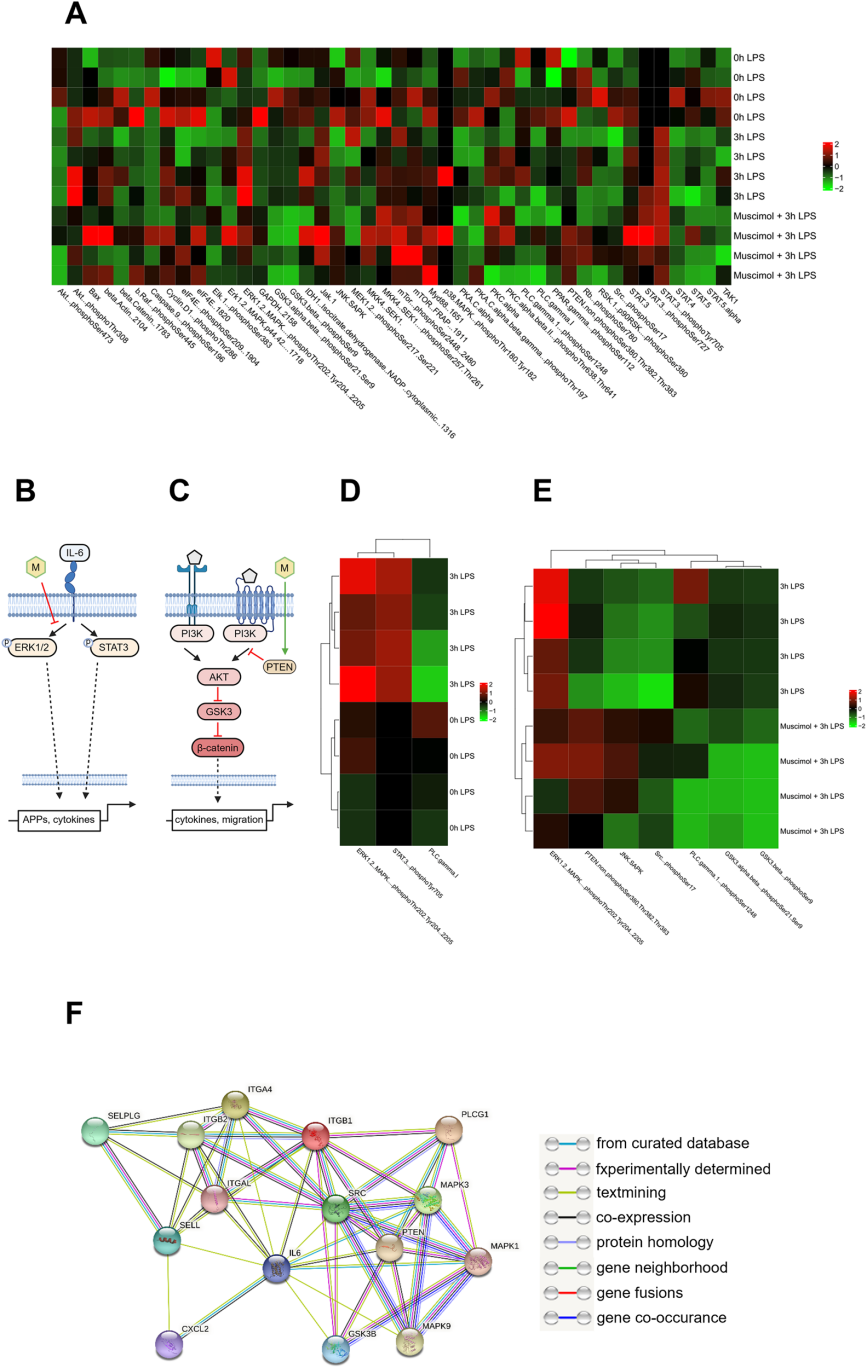
Signaling pathways that are influenced by Muscimol. To investigate which signaling pathways are affected by modulation of the GABA_A_-receptor, 44 signaling proteins were tested using DigiWest. **A** Overview of the expression of all signaling molecules investigated. Each row represents a sample of wild type animals four hours after LPS inhalation, and each column represents the normalized expression of a signaling protein. The expression level is color-coded from red (upregulated) to black, to green (downregulated). Samples from each of four animals in the control group without LPS, four hours after LPS, and in the treatment group with Muscimol were tested. **B** Schematic representation of Muscimol (illustrated as M) inhibiting the Interleukin-6 (IL-6) signaling pathway. Muscimol blocks the phosphorylation of Extracellular-signal Regulated Kinase 1/2 (ERK1/2), but not phosphorylation of signal transducer and activator of transcription 3 (STAT3). Created with biorender.com. **C** Schematic representation of Muscimol (M) inhibiting the Phosphoinositide 3-kinase (PI3K) signaling pathway. Muscimol activates Phosphatase and tensin homolog (PTEN), which leads to less AKT and more activated Glycogen synthase kinase 3 (GSK3). GSK3 inhibits β-catenin and therefore transcription. Created with biorender.com. **D** Shown are the signaling proteins with significant results of statistical analysis between the control groups without and with LPS. **E** Statistical analyses were performed by unpaired *t*-tests. Signaling proteins with statistically significant effect when comparing mice four hours after LPS with the treatment group receiving Muscimol. **F** String analysis was performed to determine the relationship between selectins, integrins, the proinflammatory cytokine IL-6 and the signaling proteins whose expression was significantly altered by Muscimol treatment.

44 signaling proteins were investigated. A heatmap for all investigated proteins is shown in Fig. 7A. IL-6 and Phosphoinositide 3-kinases (PI3K) signaling are important for inflammation and neutrophil migration and Muscimol influenced both. Binding of IL-6 activates phosphorylation of Extracellular-signal Regulated Kinases (ERK)1/2 and Signal Transducer and Activator of Transcription 3 (STAT3) (Fig. 7B). PI3K activates AKT1 and Phosphatase and Tensin homolog (PTEN) can inhibit AKT1 (Fig. 7C). LPS inhalation led to upregulation of phospho-ERK1/2 (Thr202 + Tyr204) and phospho-STAT3 (Tyr705) and the expression of PLCγ1 was inhibited (Fig. 7D). Phospho-ERK1/2 (Thr202 + Tyr204), phospho-PLCγ1 (Ser1248), phospho-GSK3αβ (Ser21 + Ser9), and phospho-GSK3β were downregulated after Muscimol and non phospho-PTEN (Ser380 + Thr382 + Thr383), JNK/SAPK and phospho-Src (Ser17) had an increased expression after Muscimol (Fig. 7E).

We hypothesized that the effects of Muscimol on IL-6, adhesion molecules and the signaling molecules are closely related. Therefore, we performed a STRING analysis, that showed both—a connection between IL-6 and the signaling proteins, and very strong connections with the integrins LFA-1 and VLA-4 (Fig. 7F).

In conclusion, the administration of Muscimol resulted in less expression of LFA-1 and VLA-4 on the surface of neutrophils and inhibited the pro-inflammatory signaling pathways IL-6 and PI3K. Additionally, the application of Muscimol resulted in a reduced release of chemoattractants and diminished expression of adhesion molecules, that can lead to the observed inhibition of migration in wild type mice.

## Discussion

The GABA_A_-receptor has been detected in the periphery such as immune cells or the lung [5, 6]. In this study, the expression of different GABA_A_-receptor subunits in the lung is shown. Comparable to our study, Jin et al. showed a low expression of receptor subunits in adult rat lungs and observed the α1 subunit to be highly expressed [14]. Furthermore, we detected the two subunits α1 and γ2 on lung epithelial cells, lung microvascular endothelium, and on neutrophils. These data are supported by various studies proving the presence of GABA_A_-receptors on lung endothelial and epithelial cells [3, 23]. The expression of the GABA_A_-receptor on neutrophils has been controversially reported. Nigam et al. detected the α1 subunit on human cells, but Sanders et al. reported that murine neutrophils do not express the GABA_A_-receptor [4, 24]. They showed that 5% of neutrophils express α1, but all other subunits were undetectable, leading the authors to assume that neutrophils do not express GABA_A_ receptors.

Our data depicted that LPS-induced inflammation increased expression of all subunits. In line with our findings, dynamic changes in the expression of the receptor subunits during an infection with influenza in human have been shown [25].

Both, GABA_A-_receptor agonist and antagonist can lead to anti-inflammatory effects [15, 18, 19, 26]. In our study, Muscimol reduced expression of all the subunits examined at the transcriptional level, while Bicuculline inhibited α1, α3, β1 and β2 expression, but increased γ2 and ε subunits. On the protein level, treatments exerted their effects on the GABA_A_-receptor on endothelial and epithelial cells.

Migration of different cell types can be influenced by the modulation of the GABA_A_-receptor. Bhandage et al. observed a reduced migration of phagocytes by antagonization of the GABA_A_-receptor during infection [27]. Chen et al. demonstrated inhibited migration of liver cancer cells and reduced metastasis by activating GABA_A_-receptors [28]. In our study, both Muscimol and Bicuculline decreased neutrophil migration in wild type animals. In the GABAα1-knock-in mice, the neutrophil migration was unaffected by both agents, indicating that they exerted their effects in an α1 subunit-dependent manner. The activity of neutrophils was not affected by the modulation of the GABA_A_-receptor, supporting the statement of Sanders et al., that diazepam, a GABA_A_-receptor agonist, exerts no effect on the function of neutrophils [24].

There are only few studies investigating the influence of the GABA_A_-receptor on migratory processes of immune cells [24]. We demonstrate that modulation of the GABA_A_ receptor affected expression of adhesion molecules on neutrophils.

TNFα was not affected by receptor modulation in wild type animals, whereas the IL-6 release was significantly reduced by both agents. Lee et al. described a reduction in the release of TNFα and IL-6 by Muscimol in vitro [26]. Two other studies described reduced serum levels of TNFα and IL-6 in rats [18, 19]. The comparability to our study is limited due to the different species.

Furthermore, we have shown that Muscimol counteracts the increased permeability after LPS while Bicuculline failed to show any effect. Tyagi et al. demonstrated reduced permeability after GABA and Muscimol, reinforcing our results [23]. Chintaragi et al. demonstrated decreased permeability in a murine lung injury model [5]. This study supports our findings that GABA_A_-receptor agonist reduced permeability and the antagonist itself has no effect.

The IL-6 and the PI3K pathway are pro-inflammatory signaling pathways. The PI3K pathway is responsible for leukocyte recruitment [29]. IL-6 has an influence on the release of acute phase proteins and regulates the migration of neutrophils [30–32]. Bell-Horner et al. described ERK1/2 as a negative modulator of GABA_A_ function, and Oscarsson et al. demonstrated propofol, an unspecific GABA_A_ agonist, to increase activation of ERK1/2 in rats [33, 34]. Yang et al. showed that electroacupuncture inhibited GABA_A_-receptor expression while reducing inflammation via the Januskinase (JAK/STAT3) pathway [35]. Our data confirmed these findings, as LPS increased the receptor subunit expressions and the activated form of ERK1/2 and STAT3. Matching the data from Yang et al., we showed a decreased expression of the subunits of the GABA_A_ receptor by Muscimol while the inflammation was inhibited by a reduced phosphorylation and thus activation of ERK1/2. The activation of STAT3 was not affected in our study.

In the PI3K signaling pathway, AKT inhibits the activation of Glycogen Synthase Kinase 3 (GSK3), resulting in increased transcription [36]. PTEN has a regulatory function regarding the influx of neutrophils [37]. It inhibits the PI3K signaling by reducing the activation of AKT, which leads to activation of GSK3 [38]. Liu et al. established a link between PTEN and the GABA_A_ receptor. Transfection with the fragile X mental retardation protein (FMRP) increased the expression of PTEN and the GABA_A_-receptor, but inhibited receptor function [39]. We showed that Muscimol upregulated the active form of PTEN and downregulated the inhibited form GSK3α and GSK3αβ, while downregulating the expression of the receptor subunits [39]. It is hypothesized that the lower expression also leads to reduced function, thereby affecting the PI3K signaling pathway.

Inhibitory Phospholipase gamma 1 (PLCγ1) was downregulated and active Src and Jun amino-terminal kinases/Stress-activated Protein Kinases (JNK/SAPK) were upregulated in our study. PLCγ1 has a key role in cell motility and Src kinases control diverse functions of neutrophils [40, 41]. Both play important roles in integrin-signaling [42–44]. JNK/SAPK is a protein of the Toll-like receptor 4 (TLR4) signaling pathway and influences the production of cytokines in inflammation [45].

We linked our results to the neutrophil surface expression of integrins, IL-6 release, and modulation of signaling proteins after Muscimol treatment in a STRING analysis. We assume that the lower surface expression of LFA-1 and VLA-4 on neutrophils inhibited the IL-6 and PI3K signaling cascades, which led to a lower release of pro-inflammatory cytokines and reduced migration of neutrophils. Various studies confirm these correlations. Velling et al. described that adhesion via β1 integrins influenced the PI3K pathway [46]. Shain et al. demonstrated that β1-integrin adhesion increases STAT3 signaling mediated by IL-6 [47]. Roy et al. observed that LFA-1 signaling increases T cell migration [48].

In summary, we identified the GABA_A-_receptor as an important player in acute pulmonary inflammation and showed that its modulation influences inflammation. Muscimol has anti-inflammatory effects on neutrophil migration and microvascular permeability. Bicuculline inhibited neutrophil migration but had no impact on permeability. Furthermore, Muscimol inhibited the IL-6, PI3K and TLR4 signaling. Abolition of the α1 subunit in GABAα1-knock-in mice abolished the anti-inflammatory effects and demonstrated the key role of this receptor subunit.

## Material and methods

### Animals

C57BL/6 J mice were purchased from Charles River Laboratories (Sulzberg, Germany) and GABAα1-Knock-in mice were imported from the Institute for Pharmacology and Toxicology of the University of Zürich, Switzerland. These mice carry the α1(H101R) point mutation which abolishes modulation of α1-GABA_A_-receptors by benzodiazepines [49], and also has hypomorphic properties (e.g., a reduced GABA sensitivity [50]). Used animals were male and between eight and 12 weeks old. All animal protocols were approved by the Animal Care and Use Committee of the University of Tübingen (A02/16 and A02/20 G) and adhered to the principles of the 3Rs (Replacement, Reduction, Refinement) to ensure ethical standards.

### Reagents

Midazolam (15 mg/kg body weight (BW), Hameln pharma plus GmbH, Hameln, Germany) was intraperitoneally (i.p.) injected one hour before LPS inhalation. 30 minutes before LPS inhalation, Muscimol (0,1 mg/kg BW, Sigma-Aldrich, St. Louis, MO, USA, i.p.), respectively Bicuculline (1 µg/kg BW, Sigma, i.p.) were administered.

### Murine models of acute pulmonary inflammation

LPS inhalation was performed as described before [51]. Mice inhaled LPS (*Salmonella enteritidis*, Sigma-Aldrich, 500 µg/ml dissolved in 7 ml sterile saline) in a custom-made chamber for 30 minutes. Klebsiella pneumoniae (43816, ATCC, Manassas, VA, USA) and Streptococcus pneumoniae (BAA-334, ATCC) were used for bacterial-induced inflammation. Mice inhaled a nebulized bacteria solution (6 × 10^7^ CFU dissolved in 7 ml sterile saline). Control mice inhaled a 0,9% NaCl solution.

### In vivo migration assay

24 hours after LPS-inhalation, neutrophil migration was determined via flow cytometry. In deep anesthesia, mice were thoracotomized, blood was collected, and PBS was injected to flush blood circulation. For bronchoalveolar lavage (BAL), a tracheal incision was conducted, PBS was installed. Lung tissue was enzymatically digested and homogenized. BAL was centrifuged and the pellet resuspended.

### Flow cytometry staining after LPS inhalation

The whole blood, lung and BAL cell suspension were incubated with CD45 (clone 30-F11, 103130, BioLegend, San Diego, CA, USA) and Ly6G (clone 1A8, 127618, BioLegend). Additionally, we evaluated the surface expression on neutrophils of CD62L (clone MEL-14, 104424, BioLegend), CD162 (clone 2PH1, 555306, BD Biosciences, Franklin Lakes, NJ, USA), CD49d (clone R1-2, 103614, BioLegend) and CD11a/CD18 (clone H155-78, 141006, BioLegend). Unless indicated otherwise sample size is given in the following order of groups: without LPS/with LPS/LPS + Muscimol/LPS + Bicuculline. For migration data of wild type mice it was 7/7/6/6 and for GABAα1-Knock-in mice 8/11/7/7. The control group without LPS is not shown in the data for the quantification of adhesion molecules due to insufficient cell numbers to perform a statistical statement. Sample size for wild type mice was 8/8/8, for GABAα1-Knock-in mice 10/8/8.

### Flow cytometry staining after bacterial inflammation

Ly6G antibody (clone 1A8, 127614, BioLegend) was injected into the tail vein before mice were anesthetized. The whole blood, lung and BAL cell suspensions were then labeled to differentiate between neutrophils attached to the endothelium (CD45^+^/Ly6G-PE/Cy7^+^/Ly6G-APC^+^) and interstitial neutrophils (CD45^+^/Ly6G-PE/Cy7^+^/Ly6G-APC^-^). The gating process is shown in SI 3. Supplementary information is available at Cell Death & Disease’s website at the end of the article and before the references. Sample size in bacterial experiments was 6/6/6/6.

### Blood gas analysis

Four hours after LPS inhalation, mice were anesthetized and connected to a ventilator (Servo 900 C; Siemens, Munich, Germany). After 30 minutes pressure-controlled ventilation at an inspiratory pressure level of 15 mbar/45 mbar a thoracotomy was performed and arterial blood was collected. Blood gases (pO_2_, pCO_2_) and lactate were measured with the ABL800 FLEX blood gas analyzer (Radiometer Medical ApS, Brönshöj, Denmark). Sample size was 7/9/6/6.

### Chemokine release

The release of chemokines was measured for CXCL1/KC (keratinocyte-derived chemokine), CXCL2/3/MIP-2 (macrophage inflammatory protein-2), TNF-α (tumor necrosis factor-α) and IL-6 in the BAL four hours after LPS inhalation with ELISA kits according to the manufacturer’s protocol (R&D Systems, Minneapolis, MO, USA). The sample size of wild type mice was 6/6/6/6 and of GABAα1-Knock-in mice 6/8/8/8.

### Gene expression

Total RNA was isolated from murine lungs using peqGold TriFast (Peqlab, Erlangen, Germany) and the iScript kit for cDNA synthesis (Bio-Rad, Munich, Germany). Both according to the manufacturer’s protocol. Real-time PCR was performed for analysis of gene expression (Primer list, SI 4). 11/13/10/10 was the sample size used for gene expression experiments.

### Microvascular leakage

Protein extravasation into the alveolar space was investigated by measuring the total protein concentration in the BAL using bicichinoninic acid (BCA) assay (Thermo Fisher Scientific, Waltham, MA, USA) three and 24 hours after LPS inhalation, according to the manufacturer’s protocol. Sample size of wild type animals was 6/6/6/6 and of GABAα1-Knock-in mice 6/6/6/6.

### Immunohistochemistry

Paraffin-embedded mouse lungs were cut into 3 µm sections. Sections were blocked and incubated with Ly6G antibody (1:500) (ab25377, abcam, Cambridge, United Kingdom) at 4 °C overnight. After incubation with biotinylated secondary antibody (1:500) (Vector Labs, Peterborough, United Kingdom) and Vectastain ABC reagent (Vector Labs), DAB chromogen was incubated (Vector Labs). Images were processed with a Leitz DM IRB microscope (Leica, Wetzlar,Germany) and AxioVision software v4.8.2. ImageJ (1.53k). *N* = 3 animals in 2 independent experiments were used for immunohistochemistry.

### Immunofluorescence

Paraffin-embedded lung sections were fixed, washed and permeabilized. After blocking, the sections were stained with primary antibodies (SI 5). To label the primary antibodies, secondary antibodies were used (SI 5). Roti-Mount FluorCare DAPI (HP20.1, Carl Roth, Karlsruhe, Germany) was used for nuclei counterstaining. (IgG-controls shown in SI 6). *N* = 3 animals in 2 independent experiments were used for immunofluorescence staining.

### Quantification of neutrophil myeloperoxidase release and NET formation

Myeloperoxidase (MPO) release was determined 24 hours after LPS inhalation in the BAL colorimetrically at 405 nm (Tecan microplate reader Infinite M200 PRO, Tecan, Männedorf, Switzerland).

NET formation was determined via double-stranded DNA (dsDNA) with the Quanti-iT dsDNA PicoGreen assay kit (P7589, Invitrogen, Carlsbad, CA, USA), according to the manufacturers’ protocols. Sample size for those experiments was 6/6/6/6.

### DigiWest

DigiWest was done as published [52]. Gel electrophoresis was performed and plotted. Proteins were biotinylated, sample lanes were cut into 96 strips. Beads were coupled and bead populations were pooled to reconstitute the original sample lane. The 44 primary antibodies were incubated overnight (SI 7). Species-specific secondary antibodies (Dianova, Hamburg, Germany) were added. Readout took place on a Luminex FlexMAP 3D instrument. Peak integration was performed using MS Excel. Signal intensities were normalized to total protein amount. R software was used for heatmap generation. For DigiWest a sample size of 4/4/4/4 was used.

### In vitro neutrophil migration assay

Human endothelial cells (HMEC-1) (CRL-3243, ATCC, Manassas, USA) were cultivated on top of a transwell insert (Corning, Corning, NY, USA), human epithelial cell (H441) (HTB-174, ATCC, USA) on the bottom. Neutrophils isolated from human whole blood migrated through the monolayer along a gradient (N-Formylmethionine-leucyl-phenylalanine (fMLP) 10 ng/ml, Sigma-Aldrich). The volunteers were informed in advance, and the sampling was approved by the ethics committee of the University Hospital of Tübingen (483/2021BO2). Either endothelial/epithelial cells or neutrophils or both were treated with Muscimol (100 µM) or Bicuculline (100 µM), one hour before stimulation with fMLP. For the quantification of migrated neutrophils, MPO was measured as described above. A sample size of 6/7/10/10 in 3 independent experiments was used.

### Statistical analysis

Data are presented as mean ± standard error of mean (SEM). Statistical analysis was performed using GraphPad Prism version 9.3 (GraphPad Software, San Diego, USA). Data were tested for outliers using the Robust regression and Outlier removal (ROUT) method at a coefficient Q = 1%. Outliers were removed. Subsequently, data were tested for normal distribution by investigating kurtosis, skewness and Q-Q plots. The required number of animals needed was based on our previous experience. No a priori statistical power calculation was conducted. The mice were assigned randomly to the groups and used in alternating sequential order, and experiments were performed at the same time points. Blinding of the experimenters took place in the animal experiments to avoid subjective bias. No *P* value adjustments were made for these interim analyses of the data. In case of normal distributed data, an independent, two-tailed Student’s *t*-test or one-way analysis of variance (ANOVA) was performed. If data was not normally distributed, Mann-Whitney-U test and Kruskal-Wallis test were used. P < 0.05 was statistically significant. All tests were two-sided.

## Supplementary information

SI 1: Statistical analysis of GABAA receptor subunits.

SI 2: Immunofluorescence staining of GABAA receptor subunits α1 and γ2.

SI 3: Gating strategy for flow cytometry-based analysis of neutrophils.

SI 4: PCR Primer list.

SI 5: Primary and secondary antibodies used for immunofluorescence staining.

SI 6: Representative images of IgG controls.

SI 6: DigiWest Primer list.

## Data Availability

The datasets generated during and/or analysed during the current study are available from the corresponding author on reasonable request.
