# Supplementary material for: Regulation of neutrophil migration in acute pulmonary inflammation by extraneuronal α1 gamma-aminobutyric acidA receptors
Source: Cell Death Dis. 2025 Apr 18;16(1):313. doi: 10.1038/s41419-025-07488-1 (PMC12008292; doi:10.1038/s41419-025-07488-1)

Supplementary Information 1: Statistical analysis of $GABA_A$ receptor subunits. The subunits A) α1, B) α3, C) β1, D) β2, E) γ2, and F) ε. Data are presented as mean ± SEM. **P* < 0.05, ***P* < 0.01, ****P* < 0.001, *****P* <0.0001. One-way ANOVA and Bonferroni correction was used for multiple group comparison.

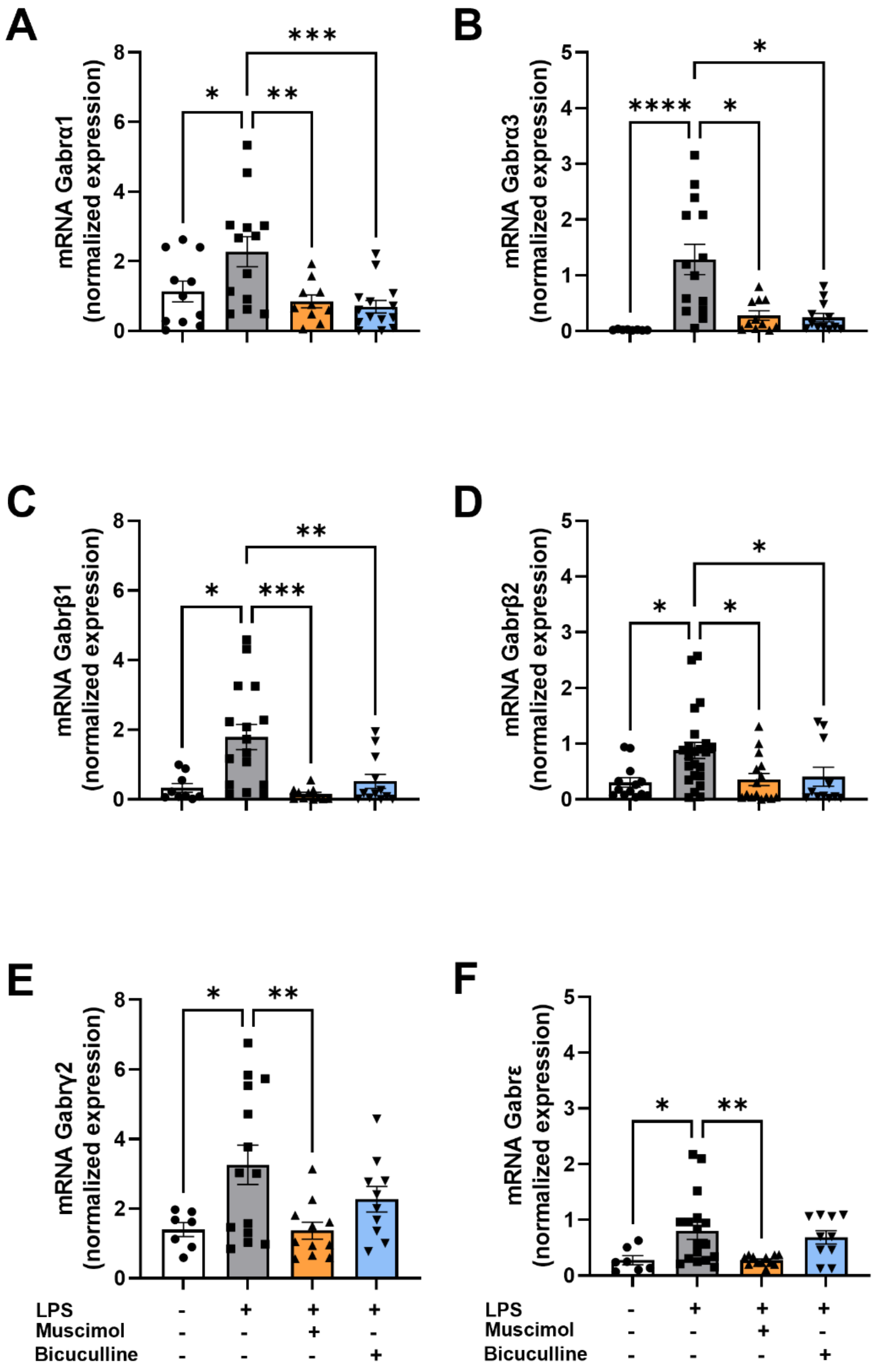

Supplement: Supplementary file 1 — SI 1: Statistical analysis of GABAA receptor subunits. [file 41419_2025_7488_MOESM1_ESM.pdf]
