# Supplementary material for: Regulation of neutrophil migration in acute pulmonary inflammation by extraneuronal α1 gamma-aminobutyric acidA receptors
Source: Cell Death Dis. 2025 Apr 18;16(1):313. doi: 10.1038/s41419-025-07488-1 (PMC12008292; doi:10.1038/s41419-025-07488-1)

Supplementary Information 2: Representative images of immunofluorescence staining of $GABA_A$ receptor subunits A) α1 and B) γ2 on lung epithelial cells, microvascular endothelial cells and neutrophils of Knock-In mice.

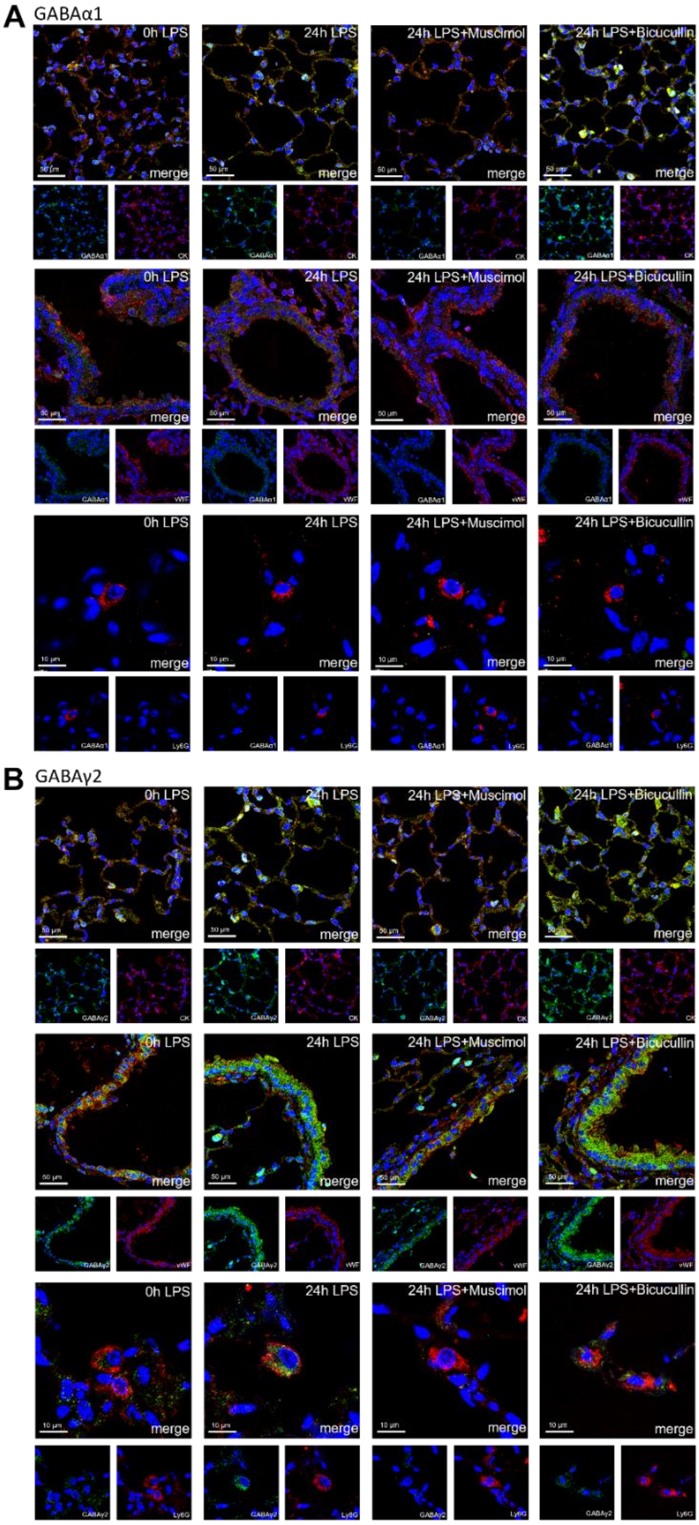

Supplement: Supplementary file 2 — SI 2: Immunofluorescence staining of GABAA receptor subunits α1 and γ2. [file 41419_2025_7488_MOESM2_ESM.pdf]
