# Supplementary material for: Regulation of neutrophil migration in acute pulmonary inflammation by extraneuronal α1 gamma-aminobutyric acidA receptors
Source: Cell Death Dis. 2025 Apr 18;16(1):313. doi: 10.1038/s41419-025-07488-1 (PMC12008292; doi:10.1038/s41419-025-07488-1)

Supplementary Information 3: Gating strategy for flow cytometry-based analysis of neutrophils using the example of a lung sample. A) Gating of potential Leukocytes based on size (FSC) and granularity (SSC). B) Gating of $CD45^{+}$ cells. C) $Ly6G^{+}$ cells were described as neutrophils. D) Differentiation between endothelial attached neutrophils ($Ly6G^{+/+}$) and interstitial neutrophils ($Ly6G^{+/-}$).

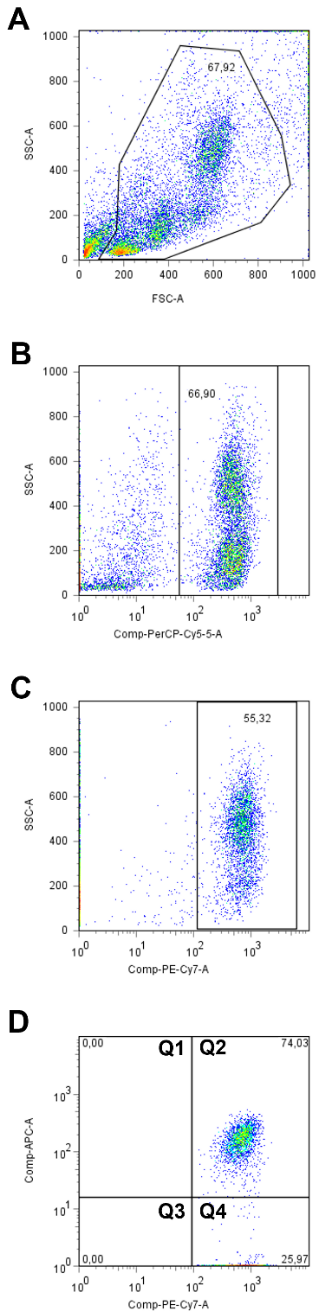

Supplement: Supplementary file 3 — SI 3: Gating strategy for flow cytometry-based analysis of neutrophils. [file 41419_2025_7488_MOESM3_ESM.pdf]
