# Supplementary material for: Regulation of neutrophil migration in acute pulmonary inflammation by extraneuronal α1 gamma-aminobutyric acidA receptors
Source: Cell Death Dis. 2025 Apr 18;16(1):313. doi: 10.1038/s41419-025-07488-1 (PMC12008292; doi:10.1038/s41419-025-07488-1)
Supplement: Supplementary file 4 — SI 4: PCR Primer list. [file 41419_2025_7488_MOESM4_ESM.pdf]

Supplementary Information 4: PCR Primer for $GABA_A$ receptor subunits with corresponding gene bank number and sequence.

Gene	Gene bank number	Sequence (5’-3’)
mGabra1	NM_010250.5	F: cac cag ttt cgg acc agt tt<br>R: agc aga gtg cca tcc tct gt
mGabra3	NM_008067.4	F: gtg aca ctc gat ctc aca ggt ct<br>R: cca tcc aaa agc cga tcc aag
mGabra4	NM_010251.2	F: tcc tgg att tgg ggg tcc tgt<br>R: gtc atc gtg agg act gtg gtt
mGabrb1	NM_008069.5	F: ccg ccg act aag ttg cat tc<br>R: tat gct ggc gac atc gat cc
mGabrb2	NM_008070.5	F: gat gat gtc gtg gag tgg tg<br>R: cct aag gcc cac atg aaa ga
mGabrb3	NM_008071.3	F: gga gga agg ctt ttc ggc at<br>R: cat gtt ccc ggg gtc gtt ta
mGabrg2	NM_008073.4	F: tct ctg ccc aag gtc tcc ta<br>R: ttg aag gtg tgt ggc att gt
mGabrg3	NM_008074.3	F: gac tgt ggt tct gtc ctg gg<br>R: tcc atc ttg tgg aat ctg gat gt
mGabre	NM_017369.2	F: tat tct gca tgg caa cgt ggt<br>R: aga gca ttg tgg tga cag aag
18S	NC_000021.9	F: gta acc cgt tga acc cca tt<br>R: cca tcc aat cgg tag cg
