# Supplementary material for: Regulation of neutrophil migration in acute pulmonary inflammation by extraneuronal α1 gamma-aminobutyric acidA receptors
Source: Cell Death Dis. 2025 Apr 18;16(1):313. doi: 10.1038/s41419-025-07488-1 (PMC12008292; doi:10.1038/s41419-025-07488-1)
Supplement: Supplementary file 5 — SI 5: Primary and secondary antibodies used for immunofluorescence staining. [file 41419_2025_7488_MOESM5_ESM.pdf]

Supplementary Information 5: Primary and secondary antibodies used for immunofluorescence staining of $GABA_A$ receptor subunits and occluding on epithelial cells, endothelial cells and neutrophils.

Primary antibodies for immunofluorescence:

rabbit anti-GABA-α1 (ab33299, abcam),

rabbit anti-GABA-α3 (NB100-61096, biotechne),

mouse anti-GABA-β1 (NBP1-48319, Novus Biologicals),

rabbit anti-GABA-γ2 (NB300-151, biotechne),

mouse anti-occludin (OC-3F10, invitrogen),

mouse anti-Cytokeratin (sc-57004, Santa Cruz),

rabbit anti-von Willebrand factor (sc-8068) and

rat anti-Ly6G (ab25377, abcam).

Secondary antibodies for immunofluorescence:

donkey anti-rabbit Alexa Fluor 488 (A32790, Thermo Fisher Scientific),

donkey anti-rabbit Alexa Fluor 546 (A10040, Thermo Fisher Scientific),

donkey anti-mouse Alexa Fluor 488 (A21202, Thermo Fisher Scientific),

donkey anti-goat Alexa Fluor 594 (A11058, Thermo Fisher Scientific),

goat anti-rabbit Alexa Fluor 647 (A21244, Thermo Fisher Scientific),

goat anti-mouse Alexa Fluor 594 (A11005, Thermo Fisher Scientific),

goat anti-rat Alexa Fluor 647 (A21247, Thermo Fisher Scientific), and

rabbit anti-mouse Alexa Fluor 633 (A21063, Thermo Fisher Scientific).
