# Supplementary material for: Regulation of neutrophil migration in acute pulmonary inflammation by extraneuronal α1 gamma-aminobutyric acidA receptors
Source: Cell Death Dis. 2025 Apr 18;16(1):313. doi: 10.1038/s41419-025-07488-1 (PMC12008292; doi:10.1038/s41419-025-07488-1)

Supplementary Information 6: Representative images of IgG controls. IgG control staining for GABAα1 on A) epithelial cells, B) endothelial cells and C) neutrophils. IgG control for GABAγ2 on D) epithelial cells, E) endothelial cells and F) neutrophils. G) IgG control staining for occluding.

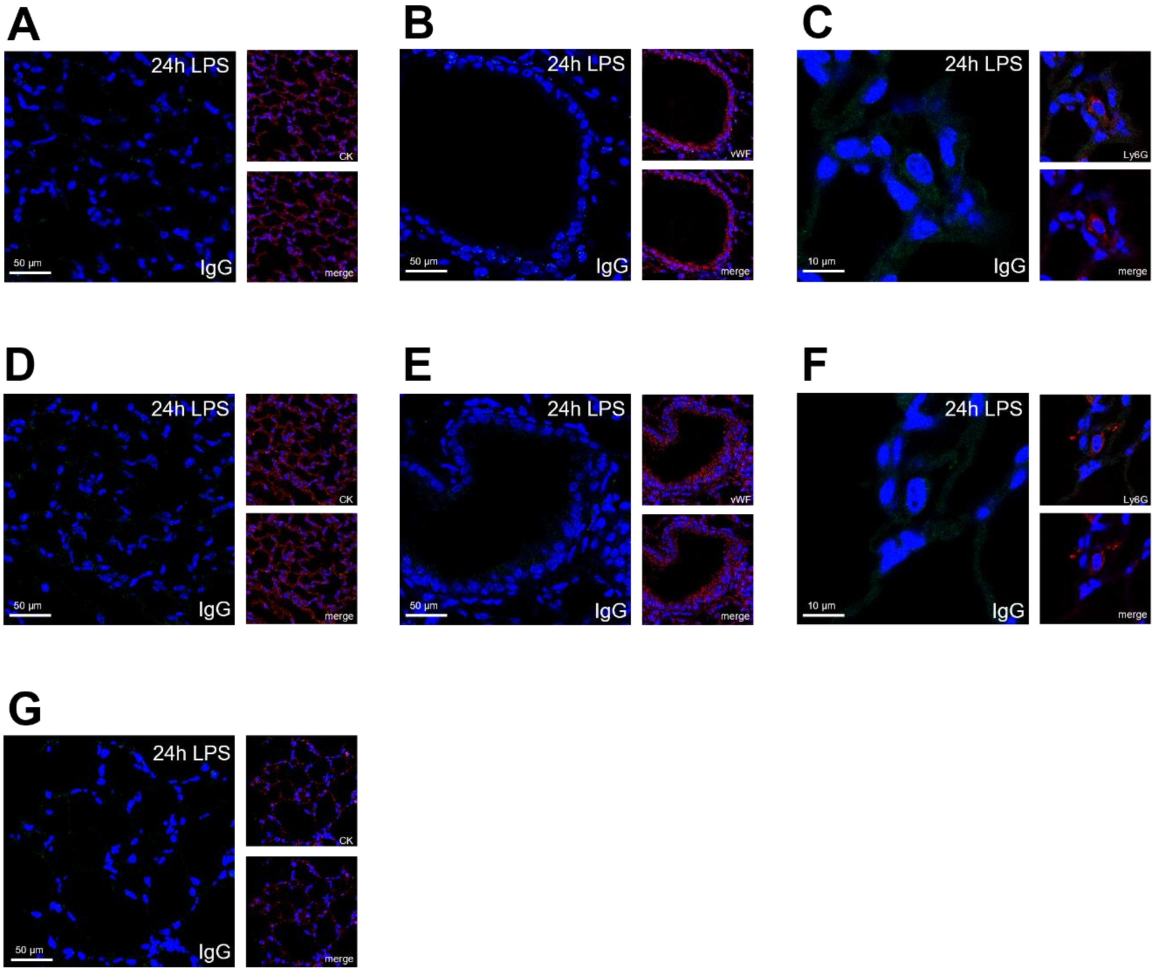

Supplement: Supplementary file 6 — SI 6: Representative images of IgG controls. [file 41419_2025_7488_MOESM6_ESM.pdf]
