# Supplementary material for: Regulation of neutrophil migration in acute pulmonary inflammation by extraneuronal α1 gamma-aminobutyric acidA receptors
Source: Cell Death Dis. 2025 Apr 18;16(1):313. doi: 10.1038/s41419-025-07488-1 (PMC12008292; doi:10.1038/s41419-025-07488-1)
Supplement: Supplementary file 7 — SI 6: DigiWest Primer list. [file 41419_2025_7488_MOESM7_ESM.pdf]

Supplementary Information 7: Primary Antibodies used for DigiWest.

Analyte Name	Supplier	Product #	Species	RRID Antibody ID
Actin beta	Sigma	A1978	ms	AB_476692
Akt - phospho Ser473	Cell Signaling	4060	rb	AB_2315049
Akt - phospho Thr308	Cell Signaling	13038	rb	AB_2629447
Bax	Cell Signaling	2772	rb	AB_10695870
beta-Catenin	Cell Signaling	8480	rb	AB_11127855
b-Raf - phospho Ser445	Cell Signaling	2696	rb	AB_390721
Caspase 9 - phospho Ser196	Thermo Fisher	PA5-40222	rb	AB_2608500
Cyclin D1 - phospho Thr286	Thermo Fisher	PA5-37487	rb	AB_2554096
eIF4E	Cell Signaling	2067	rb	AB_2097675
eIF4E - phospho Ser209	Cell Signaling	9741	rb	AB_331677
Elk-1 - phospho Ser383	Cell Signaling	9186	ms	AB_2277933
Erk1/2	Cell Signaling	4695	rb	AB_390779
Erk1/2 - phospho Thr202/Tyr204	Cell Signaling	9101	rb	AB_331646
GAPDH	Cell Signaling	5174	rb	AB_10622025
GSK3 a/b - phospho Ser21/Ser9	Cell Signaling	8566	rb	AB_10860069
GSK3 beta - phospho Ser9	Cell Signaling	9336	rb	AB_331405
IDH1	Cell Signaling	8137	rb	AB_10950504
Jak 1	Cell Signaling	3344	rb	AB_2265054
JNK/SAPK	Cell Signaling	9252	rb	AB_2250373
MEK1/2 - phospho Ser217/Ser221	Cell Signaling	9154	rb	AB_2138017
MKK4	abcam	ab33912	rb	AB_776381
MKK4 - phospho Ser257/Thr261	Cell Signaling	9156	rb	AB_2297420
mTOR (FRAP)	Cell Signaling	2983	rb	AB_2105622
mTOR (FRAP) - phospho Ser2448	Cell Signaling	5536	rb	AB_10691552
MyD88	Cell Signaling	4283	rb	AB_10547882
p38 - phospho Thr180/Tyr182	Cell Signaling	4511	rb	AB_2139682
PKA C alpha	Cell Signaling	4782	rb	AB_2170170
PKA C a/b/g - phosphoThr197	Cell Signaling	4781	rb	AB_2300165

PKC alpha	BD Biosciences	610107	ms	AB_397514
PKC a/b II-phospho T638/641	Cell Signaling	9375	rb	AB_2284224
PLC gamma I	Cell Signaling	2822	rb	AB_2163702
PLC gamma I - phospho Ser1248	Cell Signaling	8713	rb	AB_10890863
PPAR gamma - phospho Ser112	biorbyt	orb5574	rb	AB_10921220
PTEN non-phos S380/T382/383	Cell Signaling	7960	rb	AB_10971642
Rb - phospho Ser608	Cell Signaling	8147	rb	AB_10949974
RSK 1 (p90RSK) - phospho Ser380	Cell Signaling	9341	rb	AB_330753
Src - phospho Ser17	Cell Signaling	5473	rb	AB_10829921
STAT 3	Cell Signaling	4904	rb	AB_331269
STAT 3 - phospho Ser727	Cell Signaling	9134	rb	AB_331589
STAT 3 - phospho Tyr705	Cell Signaling	9145	rb	AB_2491009
